# The 20^th^ anniversary of the JACMP

**DOI:** 10.1002/acm2.12802

**Published:** 2019-12-20

**Authors:** Michael Mills

With this issue of the Journal of Applied Clinical Medical Physics, we celebrate the completion of 20 continuous years of publication and the anniversary of the publication of the first issue in the winter of 2000. I want to thank the previous Editors‐in‐Chief: Peter Almond, Ed McCullough, and George Starkschall for their years of service, building the presence, publication space, and reputation of the JACMP. I also want to recognize the continuing support of JACMP’s Deputy Editors‐in‐Chief, Tim Solberg and Per Halvorsen for their similar continuing efforts of the highest quality.

When we began in the winter of 2000, the idea of an online journal was relatively new, and the idea of an open access journal was in its infancy. The American College of Medical Physics was willing to take a chance that this new way of publishing was worth the risk, and devoted significant resources to making it successful. Also, during that era, the ACMP was successful in establishing a Board Certification process for medical physicists, and was instrumental in supporting the establishment of licensure boards for medical physicists in a few states. The American Board of Medical Physics, and licensure in New York, Texas, Florida and Hawaii, along with the JACMP, are part of the legacy of that organization. The ACMP was absorbed into the AAPM in 2012, where its spirit and its camaraderie endure.

The significance of the JACMP is not that it was an early online journal, but rather, that it was one of the first open access journals and likely the very first open access clinical medical journal. Everyone has always been able to read JACMP articles without cost through its entire existence. For sixteen of those years, authors were able to submit articles for review and publication without cost. Alas, that business model no longer works with the number of papers we are publishing, but the article publication fee (APC) for the JACMP is among the lowest in the publication industry for open access medical journals. The open access publication revolution is considered to have begun with the Berlin, Budapest, and Bethesda Declarations (https://openaccess.mpg.de/Berlin-Declaration; https://www.budapestopenaccessinitiative.org/read; http://legacy.earlham.edu/~peters/fos/bethesda.htm) in 2003. The JACMP was conceived as an open access journal in 1998 and began publication in 2000. Clearly, the vision of the ACMP was several years ahead of the international recognition of the value of open access journals.

Added to this is a rigorous peer review process that is designed to be double‐blind and strives to be fair. Our standards are high and the acceptance rate is less than 50%. Our impact factor has grown by over 50% since 2012 and now stands at a respectable 1.544. In 2019, the JACMP was associated with over 100,000 users in over 100 countries, and these numbers continue to grow. We also passed an important milestone in 2019, returning a small operating margin to the AAPM and proving the viability of the JACMP business model.

At its beginning, the JACMP targeted an open publication space. There were no journals dedicated solely to publishing the clinical applications of medical physics, and therefore some risk as to whether there would be enough articles of high quality to justify a journal. In retrospect, it seems obvious that the explosion of new imaging and radiation oncology technologies would more that justify such a periodical, but it was not so obvious to many at the time.

Consider the growth in the number of published articles over the years:

**Table:** 

Year	Published Articles	Year	Published Articles
2000	19	2010	100
2001	29	2011	103
2002	36	2012	140
2003	45	2013	166
2004	32	2014	184
2005	44	2015	193
2006	40	2016	248
2007	48	2017*	167
2008	62	2018	257
2009	80	2019	264

* 2017 reflects the beginning of the author APC charge.

The mission statement of the journal was published in the first issue and states: “…to publish papers that will help clinical medical physicists perform their responsibilities more effectively and efficiently for the increased benefit of the patient.” A quick review of the papers published over the past twenty years shows that it has lived up to this expectation, and further, it has truly become an international journal with papers being published from around the world.

I hear feedback from our education programs that JACMP articles are favored because of their easy access and lack of copyright barriers and entanglements. I am pleased that JACMP articles are favored by the medical physics education community.

Finally, congratulations to the many Associate Editors who have served and supported the JACMP over the years. You are truly the backbone of this organization and it is primarily your efforts that have built the JACMP into a journal with a strong and prominent international presence. This success belongs to you.

We are pressing forward on a number of national and international initiatives, and we look forward to seeing what the JACMP’s 25^th^ anniversary will bring.

